# Characterization of the complete mitochondrial genome of *Euwallacea fornicatus* (Eichhoff, 1868) (Coleoptera: Curculionidae: Scolytinae) and its phylogenetic implications

**DOI:** 10.1080/23802359.2020.1827070

**Published:** 2020-10-07

**Authors:** Liang-Jong Wang, Meng-Hao Hsu, Tse-Yen Liu, Ming-Ying Lin, Chia-Hsuan Sung

**Affiliations:** a Division of Forest Protection, Taiwan Forestry Research Institute, Taipei, Taiwan (R.O.C.); b Department of Plant Medicine, National Chiayi University, Chiayi City, Taiwan (R.O.C.); c Planning and Information Division, Fisheries Research Institute, Keelung, Taiwan (R.O.C.)

**Keywords:** Mitochondrial genome, *Euwallacea fornicatus*, Scolytinae, Curculionidae, next-generation sequencing

## Abstract

In the present report, we described the complete mitochondrial genome of *Euwallacea fornicatus* from Sindien, New Taipei City, Taiwan. The length of the complete mitogenome of *E. fornicatus* is 15,743 bp and the mitogenome contains 13 protein-coding, 22 tRNA and two rDNA genes. Nucleotide compositions of the whole mitogenome are 39.41% for A, 33.84% for T, 16.64% for C, and 10.11% for G. The AT and GC skewness of mitogenome sequence was 0.076 and −0.244, showing the A-skew and C-skew. The reconstructed phylogenetic relationships of 33 Curculionid species based on 13 mitochondrial protein-coding genes received absolute support (100%). *Euwallacea fornicates* is sister to the rest species in Xyleborini. The phylogenetic position of Scolytinae is sister to the clade including Cucurlioninae, Molytinae and Cryptorhynchinae. Mitogenomic data from this study will provide useful information for further studies for the population genetics, invasive history and pest control of *E. fornicatus* in the future.

The weevil subfamily Scolytinae Latreille, referred to as bark beetles, is composed of a highly diversified group of beetles with more than 6000 species assigning to 282 genera (Hulcr et al. 2015; Pistone et al. 2018). *Euwallaceae fornicates* is native to Asia but it was recently introduced to Israel, South Africa, and the United States (California) (Smith et al. 2019). It was reported that 207 tree species including avocado in Southern California were attacked by *E. fornicates* and its symbiotic fungi (Eskalen et al. 2013). The taxonomic status of the *E. fornicatus* species complex was recently studied and four species (*Euwallacea fornicatus*, *E. fornicatior*, *E. kuroshio* and *E. perbrevis*) in this confusing complex were validated by morphometric and molecular phylogenetic analyses (Stouthamer et al. 2017; Gomez et al. 2018; Smith et al. 2019). *Euwallacea fornicatus* is distributed in China, India, Japan, Malaysia, Samoa, Sri Lanka, Taiwan, Thailand and Vietnam (Smith et al. 2019). This is the first report of complete mitochondrial sequences for the species *E. fornicatus*.

The single specimen of *E. fornicatus* in this study was collected from its host plant *Wisteria sinensis* in Sindien (24°55'48.6″N; 121°30'21.6″E), New Taipei City, Taiwan, in August 2015. Total genomic DNA was extracted from the adult’s thorax using the QuickExtract™ DNA Extraction Solution kit (Epicentre, Madison, WI, USA) following the supplier’s instructions. The voucher specimen’s genomic DNA and the partial specimen (TFRIEfo001) were deposited in the Taiwan Forestry Research Institute, Taipei, Taiwan. The voucher specimen and other specimens collected in the same tree were identified to species level by L. J. Wang. The complete mitogenome of *E. fornicatus* was sequenced using the next-generation sequencing method (Illumina MiSeq, San Diego, CA)(Hahn et al. 2013). A total of 8.2 Gb next-generation sequencing paired-end reads were used to assemble the complete mitogenome sequence. The CLC Genomics Workbench (QIAGEN) was used for sequence quality analysis, data trimming, and *de novo* assembling. The locations of the protein-coding genes, ribosomal RNAs (rRNAs), and transfer RNAs (tRNAs) were predicted by using MITOS Web Server (Bernt et al. 2013) and identified by alignment with other mitogenomes of weevils. The AT and GC skew was calculated according to the following formulas: AT skew= (A – T)/(A + T) and GC skew= (G– C)/(C + G) (Perna and Kocher 1995). Maximum likelihood (ML) analyses were performed using the GTRGAMMA model implemented in RAxML v.8.1.17 (Stamatakis 2014). Nodal support confidence was estimated using a fast bootstrapping analysis with 1000 replicates in RAxML with the model GTRCAT .

**Figure 1.**
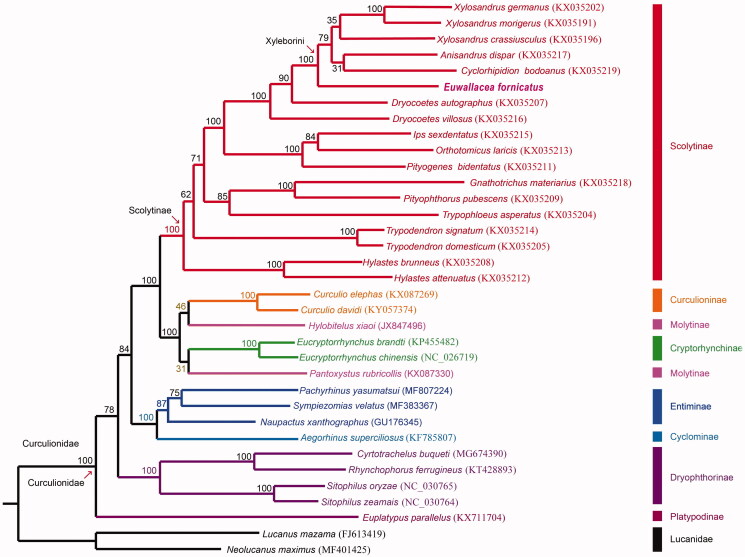
Phylogenetic tree of 33 weevil species including *Euwallaceae fornicates* (in this study, MT897842) and 2 Lucanid species based on the sequence of mitochondrial 13 protein-coding genes. The tree was reconstructed under the GTRGAMMA model implemented in RAxML v.8.1.17 (Stamatakis 2014). Nodal support confidence was estimated using a fast bootstrapping analysis with 1000 replicates in RAxML with the model GTRCAT.

The complete mitogenome of *E. fornicatus* is 15,743 bp in length (GenBank Accession No. MT897842), including 13 protein-coding genes, two rRNA genes, 22 tRNA genes and one control region. The total nucleotide composition of the *E. fornicatus* mitogenome was 39.41% for A, 33.84% for T, 16.64% for C, and 10.11% for G. The AT and GC skewness of mitogenome sequence was 0.076 and −0.244, showing the A-skew and C-skew. The gene rearrangement of the *E. fornicatus* mitogenome is similar to the inferred ancestral insect type (Cameron 2014). But, tRNA I is located in the control region (Figure 1). We reconstructed the phylogenetic relationships including 33 weevil species and two Lucanid species (*Lucanus mazama* and *Neolucanus maximus*) as outgroup based on 13 mitochondrial protein-coding genes. Bootstrap values are shown at the branch nodes. The clade including all Scolytinae species was solid supported (100%). The tribe Xyleborini including *E. fornicatus* (MT897842), *Anisandrus dispar* (KX035217)*, Cyclorhipidion bodoanus* (KX035219), *Xylosandrus crassiusculus* (KX035196), *Xylosandrus germanus* (KX035202) and *Xylosandrus morigerus* (KX035191) received absolute support (100%). *Euwallacea fornicates* is sister to the rest species in Xyleborini. Scolytinae and Xyleborini are definitely monophyletic group based on our result. The phylogenetic position of Scolytinae is sister to the clade including Cucurlioninae, Molytinae and Cryptorhynchinae in our phylogenetic result which is consistent with the results of a previous study (Zhang et al. 2019). Scolytinae and Platypodinae are not close phylogenetically. More complete mitogenomic data from other Scolytinae species are needed for further studies on the phylogeny of Scolytinae. Mitogenomic data from this study will provide useful information for further studies for the population genetics, invasive history and pest control of *E. fornicatus* in the future.

## Data Availability

The data that support the findings of this study are openly available in nucleotide database of NCBI (National Center for Biotechnology Information) at https://www.ncbi.nlm.nih.gov, accession number MT897842.
